# Tilden & Co.’s Extracts

**Published:** 1855-10

**Authors:** 


*Tilden & Co.'s Extracts.*—With the present number of the *Journal* will be found the circular of Tilden & Co. So far as we have had an opportunity to test the quality of their Extracts, we should think them as good and pure as any in the market; and consequently as worthy of the patronage of the profession.

